# Xanthogranulomatous pyelonephritis with psoas abscess and renocolic fistula

**DOI:** 10.1002/ccr3.1588

**Published:** 2018-05-29

**Authors:** Kazuya Kato, Yoshiaki Iwasaki, Yurina Kato, Kimitaka Kato, Minoru Matsuda

**Affiliations:** ^1^ Department of Surgery Pippu Clinic Pippu Town, Kamikawa‐gun Hokkaido Japan; ^2^ Department of Gastroenterology and Hepatology Okayama University Okayama City, Okayama Japan

**Keywords:** psoas abscess, renal fistula, renocolic fistula, xanthogranulomatous pyelonephritis

## Abstract

Xanthogranulomatous pyelonephritis (XGP) is an uncommon inflammatory disease of the kidney 1. Diffuse XGP is a rare condition which may spread into the pelvic cavity leading to fatal complications from a psoas muscle abscess and/or renocolic fistula 2. In diffuse type, nephrectomy and excision of the fistula is the recommended treatment.

## 1 QUESTION

**Figure 1:**
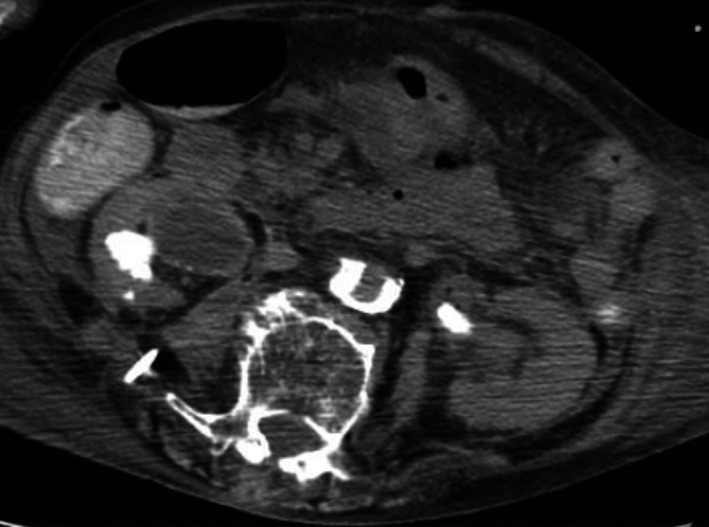
A noncontrast abdominal computed tomography (CT). An abdominal noncontrast CT revealed a marked thinning of the right renal cortex and large staghorn calculi filling the middle calyces in both kidneys

What is happening to a thinning of the right renal cortex (Figure 1)?

## 2 ANSWER

**Figure 2:**
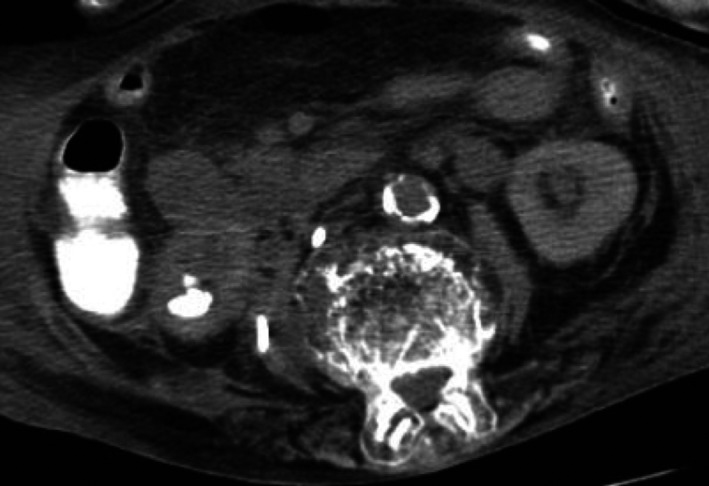
An intravenous pyelography (IVP). The IVP showed a right kidney with calculus and communication with the ascending colon

Xanthogranulomatous pyelonephritis (XGP) with a retroperitoneal abscess fistulating to the psoas muscle and the ascending colon. :

An 89‐year‐old woman presented with a fever and right flank pain. Laboratory findings revealed leukocytosis of 28 800/mm^3^. Urine analysis revealed persistent pyuria. An abdominal noncontrast CT revealed a marked thinning of the right renal cortex and a large staghorn calculi filling the middle calyces in both kidneys and a right psoas abscess (Figure 1). Percutaneous drainage of the right psoas abscess was performed. The fistulogram revealed a nephron‐psoas fistula with contrast leakage and a psoas abscess. An intravenous pyelography showed a nonvisualized right kidney with calculus and communication with the ascending colon (Figure 2). Considering the patient’s overall condition and age, she was not a candidate for surgery and was treated conservatively. The patient died 4 months after the initial admission due to DIC.

## CONFLICT OF INTEREST

None declared.

## AUTHOR CONTRIBUTIONS

KK: had full access to all of the data in the study and takes responsibility for the integrity of the data and the accuracy of the data analysis. KK: performed study concept and design. KKi: performed acquisition of data. KK, YI: performed analysis and interpretation of data, administrative, technical, or material support, and Study supervision. YK: performed drafting of the manuscript. KK, MM: performed critical revision of the manuscript for important intellectual content.
